# Anterior hyaloid membrane dissection using the conventional surgical microscope: a novel surgical approach in 2 patients

**DOI:** 10.1186/s40942-019-0191-x

**Published:** 2019-12-03

**Authors:** Radwan S. Ajlan, Joey Luvisi

**Affiliations:** 1 Department of Ophthalmology, University of Kansas School of Medicine, Kansas City, USA; 2 School of Medicine, University of Kansas, Kansas City, USA

**Keywords:** Pneumatic anterior hyaloid dissection, Anterior hyaloid membrane, Anterior proliferative vitreoretinopathy

## Abstract

**Background:**

To describe the dissection and removal of the anterior hyaloid membrane using the conventional surgical microscope.

**Case presentation:**

This microscopic surgical approach involves dissecting the anterior hyaloid at the natural anatomical plane. A 30-gauge needle mounted on a 3.0 cc syringe is used to inject filtered air anterior to the anterior hyaloid membrane. Two patients needed this procedure; the first patient was pseudophakic with proliferative diabetic retinopathy, tractional retinal detachment, and vitreous hemorrhage. The second patient was phakic with proliferative diabetic retinopathy, anterior proliferative vitreoretinopathy, and recurrent vitreous hemorrhage. Both patients tolerated the procedure well with no complications.

**Conclusion:**

Pneumatic dissection of the anterior hyaloid membrane is previously thought to be only possible with the aid of ophthalmic endoscopy. This novel surgical approach provides surgeons with the option to perform pneumatic dissection of the anterior hyaloid when ophthalmic endoscopy is not available. Prospective studies are needed to reveal possible additional benefits or risks associated with this approach.

## Background

The anterior hyaloid membrane (AHM) is a thin layer of protein fiber condensation that defines the anterior border of the vitreous body [1]. Naturally, the AHM is firmly attached to the posterior lens capsule forming the ligament of Weiger and the retrolental patellar fossa. A notable space is the canal of Petit, which is the space between the AHM, the lens, the zonules, and the pars plana epithelium [2].

The AHM becomes relevant in complex retinal detachments associated with proliferative vitreoretinopathy (PVR), where the AHM may provide a scaffold for cells to proliferate and for anterior PVR to form [3, 4]. The AHM also becomes relevant in glaucoma surgery, where residual anterior vitreous may block a pars plana placed glaucoma tube implant [5, 6].

Techniques described to remove the anterior hyaloid using the surgical microscope include direct posterior traction with the vitrector tip, or forceful injection of fluid into the anterior chamber [7, 8]. Ophthalmic endoscopy techniques for accessing the canal of Petit or the vitreous base to dissect the anterior hyaloid membrane pneumatically have certain advantages and minimize risks associated with existing microscopic techniques [9], yet ophthalmic endoscopes may not be available in all institutes, which arises the need for a novel microscopic approach to dissect the AHM. In this article, we describe a novel approach to pneumatically dissect the AHM without the use of an ophthalmic endoscope in 2 patients.

## Case description

Two patients underwent this surgical approach; the first patient was a 52-year-old pseudophakic female with poorly controlled type 2 diabetes mellitus, recurrent vitreous hemorrhage, and retinal detachment. During the surgical repair, anterior PVR was noted with heme clots staining the AHM. Microscopic pneumatic dissection of the AHM was performed using 2.0 cc of filtered air. Triamcinolone acetonide injectable suspension was injected afterward to confirm anterior hyaloid dissection and specific location of the air bubble anterior to the AHM (video 1).

The second patient was a 60-year-old phakic female who presented for recurrent vitreous hemorrhage secondary to uncontrolled proliferative diabetic retinopathy. Heme clots were present behind the natural lens and could not be reached safely with the vitrector tip; therefore microscopic pneumatic dissection of the AHM was performed and was followed by panretinal photocoagulation laser treatment, and anti-VEGF intravitreal injection.

**Fig. 1:**
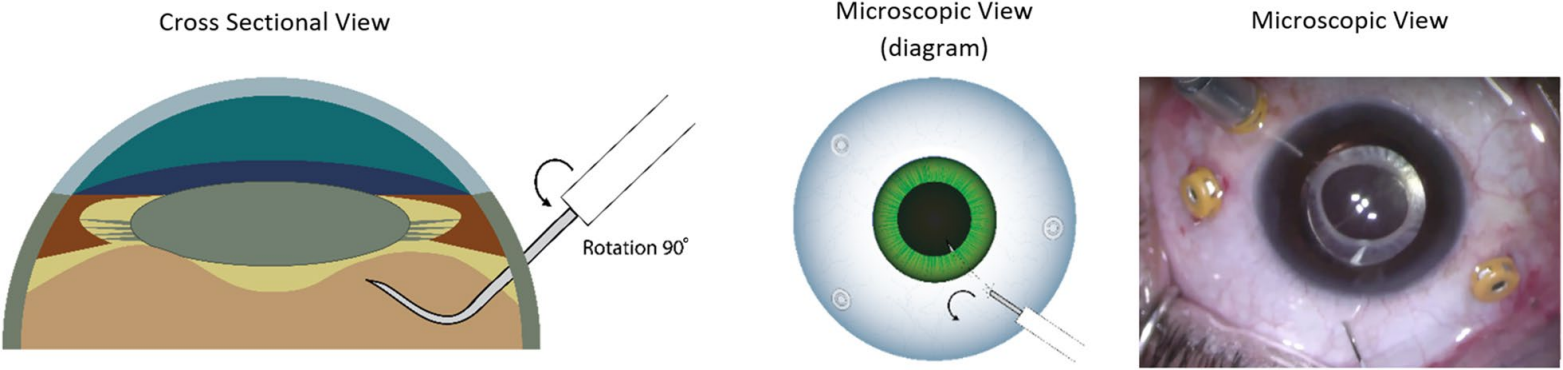
Schematic diagram with surgical view describing AHM pneumatic dissection technique showing the needle tip visualization in cross-sectional diagram view, microscopic diagram view, and actual surgical screen capture

**Fig. 2:**
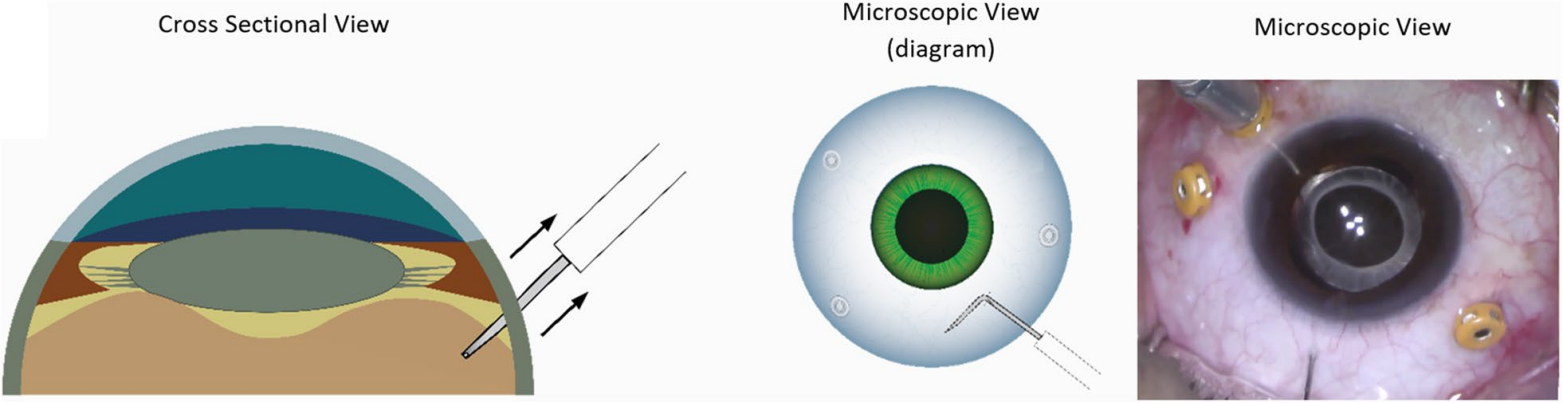
The needle tip is rotated 90° with retraction parallel to the pars plana

**Fig. 3:**
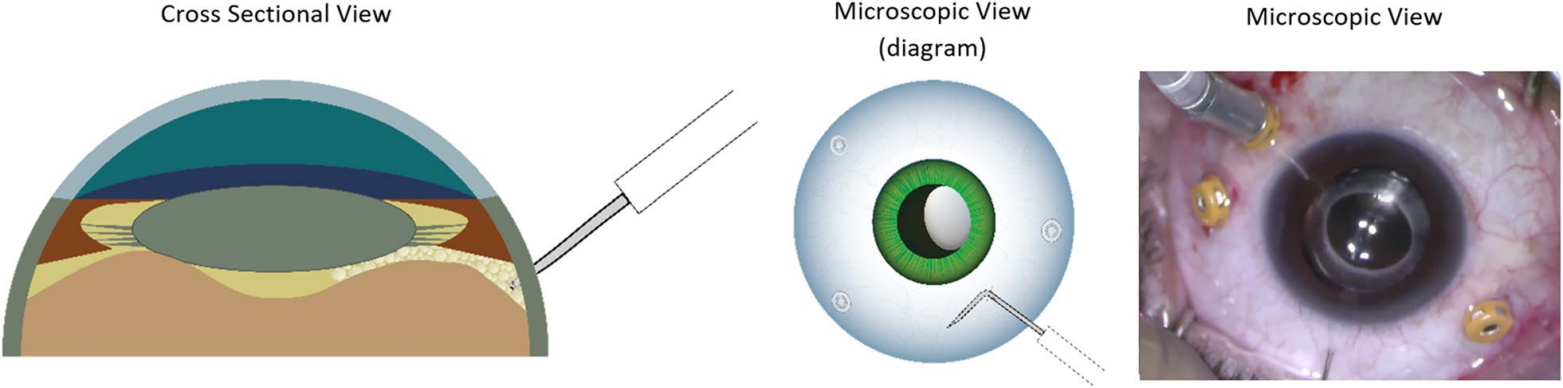
Pneumatic dissection anterior to the AHM

The surgical technique involves performing a pars plana vitrectomy (PPV) using the operating microscope, followed by careful shaving of the peripheral vitreous to minimize the risks of causing iatrogenic retinal breaks, or peripheral vitreous obstructing the infusion line cannula during pneumatic dissection. The distal 3–4 mm of a 30-gauge (G) needle is bent approximately 80° and mounted on a 3.0 cc syringe filled with filtered air. The infusion pressure is lowered to 20 mmHg to minimize intraocular pressure (IOP) spike during air injection. The needle is gently inserted through the sclera approximately 3.5 mm from the limbus. It is critical at this point to visualize the tip of the needle behind the lens to confirm entrance into the vitreous cavity before any air injection (Fig. 1). After visualizing the needle tip behind the lens, the needle is rotated 90° parallel to the limbus and gently retracted until mild resistance from the bent needle reaching the pars-plana is felt (Fig. 2). Filtered air is then injected under direct microscopic visualization (Fig. 3) (Additional file 1: Video 1). Once complete dissection of the anterior hyaloid, triamcinolone acetonide injectable suspension may be injected in the air bubble using a soft tip cannula to assure dissection of the AHM and aid in surgical plane visualization. The vitrector is then introduced to remove the dissected AHM.

## Discussion

AHM is an anatomical landmark that can be highly significant during surgical repair in patients with anterior PVR [10]. The increased anatomical success rates associated with retinectomies may be related to the release of vitreoretinal traction from residual anterior hyaloid or anterior PVR [11]. The pneumatic dissection of the AHM may help release anterior–posterior traction without the need to proceed with retinectomy or lensectomy in selected patients.

Previous microscopic techniques described to remove the anterior hyaloid include using the vitrector tip to apply manual posterior traction on the AHM [12], which may increase the risk of inducing iatrogenic retinal breaks or detachment. In addition, the injection of small air bubbles into the vitreous cavity behind the AHM may provide an estimate of the posterior lens capsule border, but does not eliminate the mechanical posterior traction induced by the vitrector tip on the anterior vitreous and possibly the lens zonules. The proposed new microscopic approach avoids direct posterior traction on the peripheral vitreous cavity or the lens zonules because the air bubble dissection occurs through the natural anatomical plane between the AHM and the lens capsule. In addition, this technique has the advantage of confirming complete dissection of the AHM, which can be difficult with other microscopic techniques.

Forceful injection of balanced saline solution (BSS) into the anterior chamber near the pupillary margin to dissect the anterior hyaloid membrane is an established microscopic technique [12]. However, the generated tension on the lens zonules cannot be eliminated and may increase the risk of lens subluxation in patients with pseudoexfoliation syndrome or weak zonules. Besides, the transparent nature of BSS can make it difficult to assure complete AHM dissection, unless there are precipitations on the AHM (e.g., blood deposits) which may provide additional visual clues. Because of the elastic nature of the AHM, when it is gets compromised by the vitrector tip, the BSS between the lens and the AHM can easily escape posteriorly into the vitreous cavity, with the residual AHM coming in direct contact with the lens again. The proposed pneumatic dissection microscopic technique addresses these factors, by injecting filtered air in the anatomical plane and minimizing tension on the lens zonules. There is a meaningful difference in the refractive indexes of the AHM and air which provides better visualization of the AHM location. In addition, when the AHM is cut by the vitrector tip, the residual air bubble remains floating, which maintains the space between the AHM and the lens.

To summarize the main advantages of microscopic AHM pneumatic dissection over available microscopic and endoscopic techniques in the literature: (1) Pneumatic dissection of the AHM spreads the dissection force through natural anatomical planes, avoiding focal traction on peripheral retinal tissue. (2) Pneumatic dissection displaces the AHM posteriorly which provides safe surgical space away from the lens and uveal tissue. (3) Injection of Triamcinolone acetonide suspension can be used to confirm the location of the air bubble. If the air bubble is posterior to the AHM, the Triamcinolone acetonide injectable suspension will be circulating through the vitreous fluid. (4) This technique minimizes the tension on the lens zonules generated from the forceful injection of BSS into the anterior chamber. (5) It can be performed in phakic and pseudophakic patients. (6) Confirmation of complete dissection of the anterior hyaloid can be achieved by direct microscopic observation. (7) While the endoscopic technique may be limited to the endoscope probe gauge size, this technique can be used with different PPV gauges (20G, 23G, 25G, and 27G).

Filtered air was chosen with this technique for the following reasons: (1) Easy visualization of the air bubble inducing the dissection. 2) The higher surface tension of the air bubble can aid in dissecting the anterior hyaloid compared to BSS. (3) The early detection of air bubbles in the anterior chamber if there is escape through the zonules when compared to BSS or a viscoelastic device (OVD). (4) The ability to use a small gauge needle (30G) with air, which may be challenging to use with OVD. (5) The ability to use Triamcinolone and confirm complete dissection of the anterior hyaloid as needed.

Risks associated with this technique involve the closely located ocular structures. If the needle tip position is not confirmed by direct visualization, air may get injected into the suprachoroidal or subretinal spaces; for this reason, direct visualization of the needle tip is essential. In addition, the needle tip insertion site needs to be through the pars plana. If the needle is inserted too anteriorly, it may cause a traumatic cataract or the ciliary body to bleed, while if the needle is inserted posterior to the pars plana, it will create a new retinal tear or detachment. In addition, air may get injected beneath the anterior hyaloid into the vitreous cavity; when in doubt, triamcinolone acetonide injectable suspension can be injected into the air bubble with injected droplets precipitating on the AHM surface if the air bubble is anterior to the AHM, or floating in the vitreous cavity if the air bubble is posterior to the AHM.

Limitations of this technique include the need for a valved trocar cannula system to create a contained pressurized system; otherwise, air bubbles will keep escaping through the non-valved trocar cannulas. Another limitation exists when extensive zonular defects or lens dehiscence are present because air bubbles can escape into the anterior chamber, which may limit the fundus view.

In conclusion, microscopic AHM pneumatic dissection through the natural anatomical planes was previously only possible with the aid of the ophthalmic endoscope. This new microscopic technique provides surgeons with the option to perform AHM pneumatic dissection when ophthalmic endoscopy is unavailable. Larger prospective studies are necessary to elucidate further potential outcomes associated with this surgical technique.

## Supplementary information

**Additional file 1: Video 1.** Conventional microscope pneumatic dissection of the anterior hyaloid membrane in a pseudophakic patient.

## Data Availability

Not applicable.

## Abbreviations

AHM: anterior hyaloid membrane
PVR: proliferative vitreoretinopathy
PPV: pars plana vitrectomy
IOP: intraocular pressure
BSS: balanced saline solution
OVD: viscoelastic device

## References

[1] American Academy of Ophthalmology. Excerpt “The Vitreous”. Clinical Education. https://www.aao.org/bcscsnippetdetail.aspx?id=8094429a-367e-436f-8859-3a1953b66d1c. Accessed 17 Feb 2019.

[2] Schachat AP (2018). Ryan’s retina, Chapter 23: vitreous and vitreoretinal interface.

[3] Lewis H, Abrams GW, Williams GA (1987). Anterior hyaloidal fibrovascular proliferation after diabetic vitrectomy. Am J Ophthalmology.

[4] Lewis H, Abrams GW, Foos RY (1987). Clinicopathologic findings in anterior hyaloidal fibrovascular proliferation after diabetic vitrectomy. Am J Ophthalmol.

[5] Desatnik HR, Foster RE, Rockwood EJ (2000). Management of glaucoma implants occluded by vitreous incarceration. J Glaucoma.

[6] Kolomeyer AM, Seery CW, Emami-Naeimi P (2015). Combined pars plana vitrectomy and pars plana Baerveldt tube placement in eyes with neovascular glaucoma. Retina.

[7] Ikeda T, Sato K, Katano T (1999). Surgically induced detachment of the anterior hyaloid membrane from the posterior lens capsule. Arch Ophthalmology.

[8] Torii H, Takahashi K, Yoshitomi F (2001). Mechanical detachment of the anterior hyaloid membrane from the posterior lens capsule. Ophthalmology.

[9] Kam YW, Funk RO, Barnard L, Ajlan RS (2019). New endoscopic surgical approach for anterior hyaloid dissection in phakic and pseudophakic patients. Retina J..

[10] Lee GD, Goldberg RA, Heier JS (2016). Endoscopy-assisted vitrectomy and membrane dissection of anterior proliferative vitreoretinopathy for chronic hypotony after previous retinal detachment repair. Retina.

[11] Quiram PA, Gonzales CR, Hu W (2006). Outcomes of vitrectomy with inferior retinectomy in patients with recurrent rhegmatogenous retinal detachments and proliferative vitreoretinopathy. Ophthalmology.

[12] Chen Y, Shah V, Jeroudi AM (2017). Surgical detachment of the anterior hyaloid membrane from the posterior lens capsule: two techniques. J Vitreo Retinal Dis.

